# An approach to difficult airway in infants: Comparison of GlideScope^®^ Spectrum LoPro, GlideScope^®^ Spectrum Miller and conventional Macintosh and Miller blades in a simulated Pierre Robin sequence performed by 90 anesthesiologists

**DOI:** 10.1371/journal.pone.0288816

**Published:** 2023-08-03

**Authors:** Andrea Irouschek, Andreas Moritz, Sven Kremer, Tobias Fuchte, Anja Danzl, Joachim Schmidt, Tobias Golditz

**Affiliations:** Department of Anesthesiology, Faculty of Medicine, University Hospital Erlangen, Friedrich-Alexander-Universität Erlangen-Nürnberg, Erlangen, Germany; Ufuk University Faculty of Medicine: Ufuk Universitesi Tip Fakultesi, TURKEY

## Abstract

**Background:**

Airway management can be challenging in neonates and infants. The Pierre Robin sequence (PRS) is a condition characterized by micrognathia, glossoptosis and airway obstruction. The airway management of these patients poses great challenges for anesthesiologists and pediatricians alike. To date, there has been no direct comparison of the hyperangulated GlideScope^®^ Spectrum LoPro (GLP), the straight GlideScope^®^ Spectrum Miller (GSM), a conventional Macintosh (MC) and a conventional Miller blade (ML) in patients with PRS.

**Methods:**

For this purpose, 90 anesthesiologists (43 with limited experience, 47 with extensive experience) performed orotracheal intubation on an Air-Sim^®^ Pierre Robin X manikin using GLP, GSM, MC and ML in randomized order. ‘Time-to-vocal-cords’, ‘time-to-intubate’, ‘time-to-ventilate’, the severity of oral-soft-tissue-trauma and the subjective evaluation of each device were recorded.

**Results:**

A significantly faster and better view of the vocal cords and lower oral-soft-tissue-trauma was achieved using the GLP (p<0.001). Though, there were no significant differences in the ‘time-to-intubate’ or ‘time-to-ventilate’. The highest intubation success rate was found with GSM and the lowest with GLP (GSM 100%, ML 97.8%, MC 96.7%, GLP 93.3%). When using the videolaryngoscopes, there were no undetected esophageal intubations but in six cases prolonged attempts of intubation (>120s) with the GLP. In the sub-group with extensive experience, we found significantly shorter intubation times for the GSM and ML. The GLP was the tool of choice for most participants, while the conventional MC received the lowest rating.

**Conclusions:**

Videolaryngoscopy leads to increased safety for the prevention of undetected esophageal intubation in the airway management in a PRS manikin. Hyperangulated blades may ensure a good and fast view of the vocal cords and low oral-soft-tissue-trauma but pose a challenge during the placement of the tube. Specific skills and handling seem to be necessary to ensure a safe tube placement with this sort of blades.

## Introduction

A difficult airway is a rare but potentially dangerous and outcome-limiting event in pediatric patients [1–5]. A single-center retrospective study included 11 219 pediatric anesthesia procedures over a 5 years period. In 1.35% of those patients, laryngoscopy was difficult, which was defined as Cormack-Lehane (CL) view III and IV [1]. These findings are supported by current data of the NECTARINE network, describing an incidence of 5.8% difficult intubations in 4683 cases, defining difficult intubations as two failed attempts using direct laryngoscopy [2, 3]. In recent years, the management of the difficult airway has been significantly simplified through the introduction of videolaryngoscopes in adult [6] and pediatric patients [7, 8].

Various predictors have been identified to anticipate a difficult airway situation, including individual anatomical, functional and morphometric parameters such as a small mandibular space, restricted head extension, increased tongue size, craniofacial dysmorphism and anatomical anomalies [9]. Furthermore, less than one year of age, body weight below the 10th percentile and an American Society of Anesthesiologists (ASA)-classification of III/IV as well as various congenital syndromes such as Treacher-Collins-syndrome, Goldenhar-syndrome, and Pierre-Robin-sequence (PRS) are associated with a difficult laryngoscopy or intubation [1, 10, 11].

PRS is a rare congenital syndrome [12], which is characterized by a triad of micrognathia, glossoptosis and airway obstruction. Securing the airway in children suffering from PRS can be challenging even for experienced anesthesiologists [13, 14].

Intubation manikins are essential to obtain the necessary routine for airway management in such rare cases and provide training opportunities for all staff. Not only the use of videolaryngoscopy but also the selection of the corresponding blade contributes significantly to the success of the intubation. In our preliminary study on a PRS manikin, we were able to find an advantage for the straight Miller blade with a Storz C-MAC^®^ videolaryngoscope with an 8-inch monitor over a curved GlideScope^®^ Spectrum LoPro blade with a GlideScope^®^ videolaryngoscope with a 10-inch monitor or a conventional Miller blade [15]. This stationary setup simulates an environment as you would find it in an operating room. However, physicians of different medical fields must be well-prepared for encountering a difficult airway situation in pediatric patients. There is a need for additional evidence on portable videolaryngoscopic solutions, as provided in intensive care units, emergency departments or prehospital emergency services.

Therefore, the aim of this prospective crossover study was the comparison of the GlideScope^®^ Go^™^ videolaryngoscope with a single-use GlideScope^®^ Spectrum Miller blade or a single-use GlideScope^®^ Spectrum LoPro blade and a conventional single-use Miller blade or a conventional single-use Macintosh blade concerning intubation success and intubation times as well as the subjective evaluation of the different laryngoscopic devices in a PRS manikin.

## Materials and methods

### Study design and setting

The local institutional ethics committee approved the here presented crossover manikin study (Ethics Committee of the Friedrich-Alexander-University Erlangen-Nürnberg; Reference number: 408_18 B). All 90 participants gave their written and informed consent prior to entering the study. The data of the participants were anonymised and information on individual performances was unavailable to anyone outside of the research team.

The study was performed on a difficult infant airway manikin (Air-Sim^®^ Pierre Robin X manikin, TruCorp Ltd, Lurgan, Northern Ireland). Each participant performed endotracheal intubation with the GlideScope^®^ Go^™^ handheld system, using a single-use hyperangulated GlideScope^®^ Spectrum LoPro S1 blade (‘GLP’) and a straight GlideScope^®^ Spectrum Miller S0 blade (‘GSM’) (Verathon Medical Canada ULC, Burnaby, BC, Canada). Additionally, each participant was asked to use a conventional Miller laryngoscope blade size 0 (‘ML’) (Rüsch^®^ Polaris Single-Use Laryngoscope Blade ML 0, Teleflex Medical Ltd, Athlone, Ireland) and a Macintosh laryngoscope blade size 1 (‘MC’) (Rüsch^®^ Polaris Single-Use Laryngoscope Blade MC 1, Teleflex Medical Ltd, Athlone, Ireland), both in combination with a Heine F.O. SLIM LED metallic laryngoscope handle (Heine Optotechnik GmbH & Co. KG, Gilching, Germany). The order of the blades used for intubation was randomized for each participant using four sealed opaque envelopes each containing the name of one of the four different blades. The participants drew the envelopes in sequence to determine the order of the blades directly prior to the start of the examination [15].

All intubations were performed with a 3.5mm uncuffed endotracheal tube (‘ETT’; pediatric soft endotracheal tube, inner diameter (I.D.) 3.5mm, outer diameter (O.D.) 5.2mm, Vygon, Aachen, Germany). A 2.6mm outer diameter Rüsch^®^ reusable intubation stylet (Teleflex Medical Ltd, Athlone, Ireland) was used in each intubation. When used with hyperangulated GLP, the study leader preformed the stylet using a former plate, which was built according to the curve of the small GlideRite^®^ stylet small (GliteRite^®^ Single-Use Stylet, Verathon Medical Canada ULC, Burnaby, BC, Canada). While using the other blades, the participants preformed the stylet at their preference.

### Participant population

Anesthesiologists from the Department of Anesthesiology of the University Hospital Erlangen, a third-level center with more than 30.000 anesthesia procedures per annum, were included in this investigation. The participants’ characteristics, the number of intubations of adults and children under 5 years of age and the number of videolaryngoscopic intubations were documented. For exact documentation, data were extracted from the electronic patient data management system (NarkoData, IMESO, Hüttenberg, Germany). The data are presented as median with inter-quartile range (IQR).

### Measurements

#### Objective findings

Intubation time was measured for each attempt and device. To compare the different devices, we defined three points of time:

1. The time to visualize the glottis (‘time-to-vocal-cords’) was defined as the time from insertion of the blade between the teeth until the glottis was visualized.
2. The time to tracheal intubation (‘time-to-intubate’) was defined as the time from insertion of the blade between the teeth until the ETT was deemed to be positioned correctly by each participant.
3. The time to ventilation (‘time-to-ventilate’) was defined as the time between the insertion of the blade between the teeth until the ETT was connected to a self-inflating resuscitation bag and lung inflation was confirmed.

The primary endpoint was the ‘time-to-intubate’. Esophageal intubations, attempts of more than 120 seconds or more than two attempts of intubation (complete withdrawal of the device from the mouth and repositioning) were recorded as failure to intubate. In the event of an undetected esophageal intubation, the intubation attempt was stopped and no further attempt was allowed. As no ‘time-to-intubate’ or ‘time-to-ventilate’ could be recorded, esophageal intubations were not included for statistical analysis of the intubation times. If no tracheal intubation was achieved within 120 seconds, the ‘time-to-intubate’ and/or the ‘time-to-ventilate’ was regarded as 121 seconds.

To avoid any interobserver bias, stopwatch studies were performed by a single member of the research team. We recorded the rate of successful intubations, the number of intubation attempts, the number of optimization maneuvers (readjustment of the head position, application of external laryngeal pressure and assistance by a second person), the severity of potential oral-soft-tissue-trauma (0 = none, 1 = mild: contact between the blade and the upper gum line, 2 = moderate: the blade bent the upper gum line, 3 = severe: the blade bent the upper gum line and the upper lip) and the laryngeal view using the CL-score [16].

#### Subjective findings

After completing the procedure, each participant was asked to score the view on the vocal cords, the handling, the stability, the force applied during intubation and the overall difficulty of tracheal intubation for each device. Therefore, we used a numeric rating scale from 0 (‘excellent/very easy’) to 10 (‘very poor/very difficult’). After completing all four intubations, the participants were asked to bring the intubation devices in the order of their preference (1 = most preferred device, 4 = least preferred device).

#### Data analysis

Before the study, a power analysis using G*Power (Version 3.1.9.4, Paul F., 2019, Germany) was conducted. Based on the experiences of previous studies, an effect size of 0.4 was anticipated. Considering an α-error of 0.05 and a β-error of 0.1, the sample size calculation indicated a total group size of at least 88 participants. All statistical analyses were performed using SPSS software (IBM^®^ SPSS, Version 28.0, IBM Corp., Armonk, NY, USA). To analyze the underlying study population, descriptive statistical analysis was used. The rate of successful intubation was compared using a Cochran’s Q test with a Dunn-Bonferroni post-hoc test. The different time points (‘time-to-vocal-cords’, ‘time-to-intubate’, ‘time-to-ventilate’), the number of intubation attempts, the number of optimization maneuvers, the severity of oral-soft-tissue-trauma as well as the subjective ratings were compared using the Friedmann test, posthoc Wilcoxon signed-rank test with a Bonferroni correction for multiple comparisons. Group-dependent differences were compared using the Mann-Whitney-U test. The data are presented as median and interquartile range (IQR). The level of statistical significance was set to p<0.05.

## Results

### Participant characteristics

We were able to include 90 anesthesiologists in this randomized crossover trial. According to the participants’ experience in clinical anesthesia, we formed two sub-groups for further statistical analysis: In the subgroup of ‘limited experience’ 43 residents were included. The sub-group with ‘extensive experience’ included 47 specialists and residents with 6 or more years of experience in clinical anesthesia. Table 1 shows detailed characteristics of the participants such as exact gender distribution, age, years of clinical experience, total number of intubations performed, videolaryngoscopic intubations and intubations in children under 5 years of age.

**Table 1:** Participant characteristics.

		sub-group analysis	
	Participants, overall (N = 90)	Anesthesiologists with limited experience in clinical anesthesia^#^ (N = 43)	Anesthesiologists with extensive experience in clinical anesthesia^##^ (N = 47)
Gender *			
Female	**37/90 (41.1%)**	21/43 (48.8%)	16/47 (34.0%)
Male	**53/90 (58.9%)**	22/43 (51.2%)	31/47 (66.0%)
Age (y) ^$^	**34.5 (31.0–42.0)**	31.0 (27.00–33.0)	41.0 (37.0–47.0)
Clinical experience (y)	**6.0 (2.0–14.6)**	2.0 (0.7–3.0)	14.5 (7.0–18.0)
Emergency medicine expertise (n)	**46/90 (51.1)**	6/43 (14.0)	40/47 (85.1)
Total number of videolaryngoscopic intubations ^$^	**48 (19.8–77.5)**	20 (9–36)	77 (54–104)
Total number of anesthesia procedures ^$^	**1701 (681–3867)**	678 (299–1019)	3765 (2226–6214)
Number of self-performed anesthesia procedures in children under 5 years of age ^$^	**66 (28.5–206.8)**	27 (3–54)	172 (89–393)

The data are presented as median (inter-quartile range, IQR) or as fraction n/N (%).

^#^ residents with less than 6 years of experience in clinical anesthesia

^##^ specialists and residents with 6 or more years of experience in clinical anesthesia

* no statistically significant difference in gender distribution (p = 0.109)

^$^ statistically significant difference between the two sub-groups (p< 0.001)

### Intubation findings

Comparing the primary endpoint ‘time-to-intubate’ and ‘time-to-ventilate’ within the group of all 90 anesthesiologists no significant difference between the devices was revealed (‘time-to-intubate’ p = 0.902; ‘time-to-ventilate’ p = 0.348). But the GLP enabled a highly significantly faster ‘time-to-vocal-cords’ compared to the other devices (p<0.001). The time to visualize the vocal cords using the GSM was also significantly shorter compared to the conventional blades (GSM vs. MC and GSM vs. ML p<0.05). There were no statistically significant differences between ML and MC.

The GLP led not only to a faster view on the vocal cords but also to a better view on the glottis scored by the CL-score (GLP vs. GSM, MC, ML p<0.001). The GSM was superior to the conventional blades (MC p<0.001, ML p = 0.037).

Regarding the oral-soft-tissue-trauma, the GLP revealed highly significant advantages compared to the other devices (p<0.001). No moderate to severe oral-soft-tissue-trauma was reported. Mild trauma occurred in only two cases. There were no differences between the other three blades. There were no differences in the number of optimization maneuvers between the blades. The number of intubations causing a deformation of the intubation stylet was significantly higher when using the GLP compared to the other three devices (p<0.001). The statistical results of intubation times and objective measurements are presented in Table 2.

**Table 2:** Detailed analysis of objective and subjective results for all participants.

	Miller-Blade	Macintosh-Blade	GlideScope Miller	GlideScope LoPro
Overall success rate, n/N (%)	88/90 (97.8%)	87/90 (96.7%)	90/90 (100%)	84/90 (93.3%)
Esophageal intubation, n/N (%)	2/90 (2.2%)	3/90 (3.3%)	0/90	0/90
Prolonged intubation (>120s; two attempts), n (%)	0/90	0/90	0/90	6/90 (6.7%)
Time-to-vocal-cords (s), median (IQR)	11.1 (5.6–17.1) *^,^^§^	10.4 (7.3–16.0) *^,^^§^	8.4 (5.9–12.8) *^,^^§^	3.9 (3.1–5.5) *
Time-to-intubate (s), median (IQR)	17.4 (12.6–27.1)	17.6 (12.6–26.8)	18.1 (12.8–25.4)	21.1 (12.4–41.2)
Time-to-ventilate (s), median (IQR)	25.1 (18.7–34.7)	24.3 (18.9–35.2)	26.6 (20.3–36.2)	29.0 (19.2–50.9)
Number of intubation attempts, n/N (%)				
1	89/90 (98.9)	89/90 (98.9)	89/90 (98.9)	87/90 (96.7)
2	1/90 (1.1)	1/90 (1.1)	1/90 (1.1)	3/90 (3.3)
3 or more	0/90	0/90	0/90	0/90
Median	1.0	1.0	1.0	1.0
Severity of oral-soft-tissue-trauma, n/N (%)				
None	18/90 (20.0)	30/90 (33.3)	22/90 (24.4)	88/90 (97.8)
Mild	61/90 (67.8)	58/90 (64.4)	60/90 (66.7)	2/90 (2.2)
Moderate	11/90 (12.2)	2/90 (2.2)	8/90 (8.9)	0/90
Severe	0/90	0/90	0/90	0/90
Median	1.0*	1.0*	1.0*	0.0*
Number of optimization maneuvers, n/N (%)				
0	67/90 (74.4)	57/90 (63.3)	73/90 (81.1)	67/90 (74.4)
1	22/90 (24.4)	33/90 (36.7)	17/90 (18.9)	14/90 (15.6)
2 or more	1/90 (1.1)	0/90	0/90	9/90 (10.0)
Median	0.0	0.0	0.0	0.0
Stylet Deformation n/N (%)				
None	88/90 (97.8)	87/90 (96.7)	87/90 (96.7)	55/90 (61.1)
Mild	2/90 (2.2)	3/90 (3.3)	3/90 (3.3)	18/90 (20.0)
Severe	0/90	0/90	0/90	9/90 (10.0)
Median	0.0*	0.0*	0.0*	0.0*
View, median (IQR)	3.0 (1.5–5.0)**	4.0 (2.0–5.3)**	2.0 (0.5–3.0)**^,^ ^$^	0.5 (0.0–1.0)**^,^ ^$^
Handling, median (IQR)	3.0 (2.0–5.0)**^,^ ^§^	3.0 (1.5–5.0)^§^	2.0 (1.0–5.0) ^§^	2.0 (1.0–4.0)**^,^ ^§^
Stability, median (IQR)	4.0 (2.0–6.0)**	4.0 (2.0–5.5)**	2.0 (1.0–4.0)**	1.0 (0.9–2.0)**
Force applied during intubation attempt, median (IQR)	1.0 (0.0–2.0)	1.0 (0.0–2.0)	1.0 (0.5–2.0)	1.0 (0.0–2.0)
Overall difficulty of intubation, median (IQR)	3.8 (2.0–6.0)^§^	4.0 (2.5–6.0)^§^	2.8 (1.5–4.0)^§^	3.0 (1.9–5.0)^§^
Cormack-Lehane Score, n/N (%)				
1	34/90 (37.8)	27/90 (30.0)	57/90 (63.3)	82/90 (91.1)
2	49/90 (54.4)	52/90 (57.8)	29/90 (32.2)	8/90 (8.9)
3	5/90 (5.6)	11/90 (12.2)	4/90 (4.4)	0/90
4	2/90 (2.2)	0/90	0/90	0/90
median	2.0 **^,^ ^§^	2.0 **	1.0 **^,^ ^§^	1.0 **

Data are presented as median (inter-quartile range, IQR), number n (%) or as fraction n/N (%). The subject findings are presented as a numerical rating scale (0 to 10, from excellent/very easy to poor/very difficult).

* p<0.001 GildeScope LoPro vs GlideScope Miller vs Miller vs Macintosh

** p<0.001 GlideScope LoPro or GlideScope Miller vs Macintosh or Miller

^$^ p<0.001 GlideScope LoPro vs GlideScope Miller

^§^ p<0.05 GlideScope LoPro or GlideScope Miller vs Miller or Macintosh

In the sub-group analysis the use of the GSM and the ML in the group with extensive experience led to a significantly faster ‘time-to-vocal-cords’ (p<0.05), ‘time-to-intubate’ (p<0.05) and ‘time-to-ventilate’ (p<0.05). For further details of the subgroup analysis, see Table 3.

**Table 3:** Sub-group analysis, anesthesiologists with limited experience vs. extensive experience in clinical anesthesia.

	Anesthesiologists with limited experience in clinical anesthesia^#^ (N = 43)	Anesthesiologists with extensive experience in clinical anesthesia^##^ (N = 47)
GlideScope^®^ Spectrum Miller		
Time-to-vocal-cords*	10.0 (6.4–16.7)	7.7 (5.7–10.8)
Time-to-intubate*	21.5 (16.4–31.5)	15.3 (11.8–22.6)
Time-to-ventilate*	30.4 (22.9–41.5)	23.7 (19.1–30.4)
Maneuvers for optimization**	0 (0–1)	0 (0–0)
Oral-soft-tissue-trauma	1 (1–1)	1 (0–1)
Deformation of the stylet	0 (0–0)	0 (0–0)
Failed intubation rate, n/N (%)	0/43 (0%)	0/47 (0%)
Conventional Miller		
Time-to-vocal-cords*	13.9 (9.5–22.0)	9.8 (6.9–14.6)
Time-to-intubate*	23.5 (15.3–29.9)	15.4 (11.8–21.7)
Time-to-ventilate*	30.6 (21.4–36.5)	21.1 (17.5–29.3)
Maneuvers for optimization*	0 (0–1)	0 (0–0)
Oral-soft-tissue-trauma	1 (1–1)	1 (1–1)
Deformation of the stylet	0 (0–0)	0 (0–0)
Failed Intubation rate, n/N (%)	2/43 (4.7%)	0/47 (0%)
Esophageal intubation, n/N (%)	2/43 (4.7%)	0/47 (0%)
Prolonged intubation (>120s), n/N (%)	0/43 (0%)	0/47 (0%)
GlideScope^®^ Spectrum LoPro		
Time-to-vocal-cords	4.5 (3.3–6.4)	3.7 (2.9–4.8)
Time-to-intubate	23.2 (13.8–39.1)	16.6 (10.6–45.9)
Time-to-ventilate	31.4 (20.8–49.6)	23.5 (18.5–54.7)
Maneuvers for optimization	0 (0–0)	0 (0–0)
Oral-soft-tissue-trauma	0 (0–0)	0 (0–0)
Deformation of the stylet	0 (0–1)	0 (0–1)
Failed intubation rate, n/N (%)	3/43 (7.0%)	3/47 (6.4%)
Esophageal intubation, n/N (%)	0/43 (0%)	0/47 (0%)
Prolonged intubation (>120s), n/N (%)	3/43 (7.0%)	3/47 (6.4%)
Conventional Macintosh		
Time-to-vocal-cords	12.4 (8.2–17.3)	9.5 (6.8–14.8)
Time-to-intubate	19.9 (15.1–32.4)	16.4 (11.2–25.2)
Time-to-ventilate	26.3 (20.8–39.0)	23.2 (17.8–32.8)
Maneuvers for optimization*	0 (0–1)	0 (0–1)
Oral-soft-tissue-trauma	1 (0–1)	1 (0–1)
Deformation of the stylet	0 (0–0)	0 (0–0)
Failed intubation rate, n/N (%)	1/43 (2.3%)	2/47 (4.3%)
Esophageal intubation, n/N (%)	1/43 (2.3%)	2/47 (4.3%)
Prolonged intubation (>120s), n/N (%)	0/43 (0%)	0/47 (0%)

The data are presented as median (inter-quartile range, IQR) or as fraction n/N (%).

^#^ residents with less than 6 years of experience in clinical anesthesia

^##^ specialists and residents with 6 or more years of experience in clinical anesthesia

* statistically significant difference between the groups (p< 0.05)

** highly statistically significant difference between the groups (p< 0.001)

### Intubation success rate

The overall rate for the success of intubation of the 90 anesthesiologists was 96.9% (349/360 intubations). In the subgroup with ‘limited experience’ the intubation success rate was 96,5% (166/172) and in the subgroup with ‘extensive experience’ 97.3% (183/188). The GSM led to a 100% success rate for intubation. No prolonged attempts or esophageal intubations were recorded. Using the GLP, six participants failed the intubation due to prolonged time for intubation (>120s) (‘limited experience’ 3/43 (7.0%), ‘extensive experience’ 3/47 (6.4%)). No esophageal intubations were recorded. The overall success rate for the GLP was 93.3% (84/90). Using the conventional MC three participants placed the tube in the esophagus (‘limited experience’ 1/43 (2.3%), ‘extensive experience’ 2/47 (4.3%)). The overall success rate was 96.7% (87/90). While handling the ML two anesthesiologists placed the tube esophageal (‘limited experience’ 2/43 (4.7%)). No prolonged intubations were recorded with the conventional blades. The overall success rate was 97.8% (88/90). The differences between the devices were not statistically significant (p = 0.064). For further details, see Tables 2 and 3.

### Subjective analysis

All 90 participants filled out the questionnaire completely and correctly and therefore 90 questionnaires could be included in the analysis. The detailed results from the analysis are displayed in Table 2. The view of the vocal cords using a video-based tool for intubation was rated highly significantly better than using a conventional tool (GLP or GSM vs. MC or ML, p<0.001). The GLP was rated better than the GSM (p<0.001). The rating between the MC and the ML blade did not differ significantly. Concerning the handling, the GLP was rated better than the MC (p<0.05) and the ML (p<0.001). The GSM was rated superior to the ML (p<0.05). The stability of the two video-based blades during intubation was rated highly significantly better than that of the two conventional blades (GLP or GSM vs. MC or ML, p<0.001), without a significant difference between the two video-based and conventional tools, respectively. No differences between all four devices were reported concerning the force applied during intubation. The overall difficulty was rated as follows: The difficulty using the GSM was rated lower compared to the MC (p = 0.007) and the ML (p = 0.026), same with the GLP compared to the MC (p = 0.015), but not for the ML (p = 0.056). Between the two video-based blades and the two conventional blades was no significant difference respectively.

**Fig 1:**
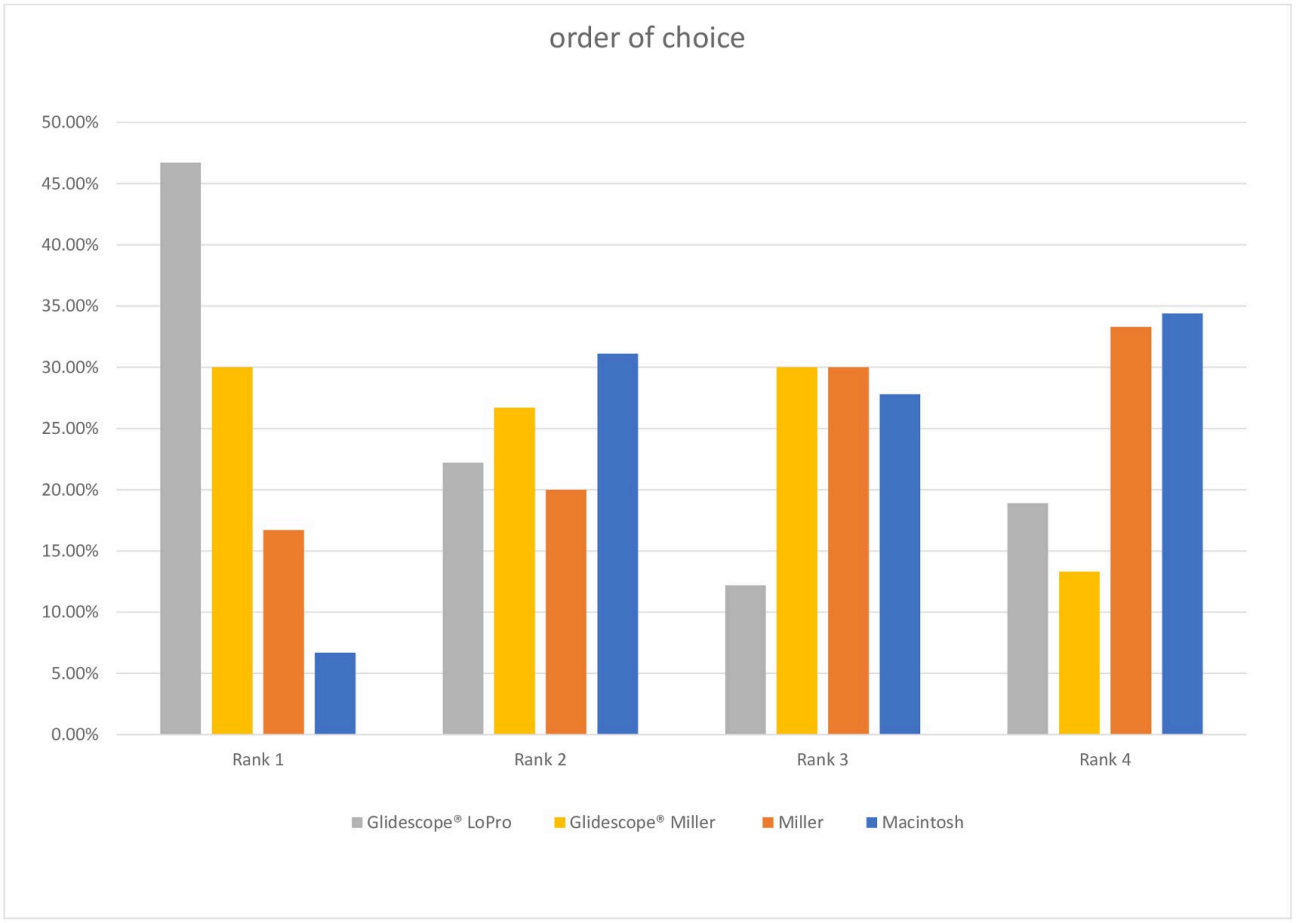
Laryngoscopes in order of choice. The participants were asked to bring the devices in an order of their preference with rank 1 being the most preferred and rank 4 being the least preferred device.

At the end of the questionnaire, the clinicians were asked to bring the devices in the order of their preference. Most clinicians (42/90, 46.7%) chose the GLP as their device of choice and the MC was chosen as the least favored device by the most (31/90, 34.4%). For detailed results please be referred to Fig 1.

## Discussion

Neonates and infants are at higher risk for respiratory and cardiovascular complications during anesthesia induction and tracheal intubation than older children. The incidence of hypoxemia with subsequent bradycardia during intubation is significantly increased due to low functional residual capacity, small closing capacity, and higher oxygen consumption [2, 3, 17]. Difficult laryngoscopy and intubation can occur more frequently in neonates and infants, even in the absence of risk factors for a difficult airway and is often difficult to anticipate preoperatively [2, 3, 17].

In an infantile difficult airway situation, the early use of videolaryngoscopy and the timley switch to alternative techniques to secure the airway in case of failed tracheal intubation with a conventional laryngoscope can make a decisive contribution to avoiding cardiorespiratory complications. It is crucial to avoid repeated attempts at intubation in patients of this age group [2, 3, 17–19].

In the presented study, two videolaryngoscope blades (GlideScope^®^Go^™^ with GLP and GSM) were compared with two conventional laryngoscope blades (Miller and Macintosh) with regard to intubation success, intubation times and subjective evaluation in a simulated difficult infant airway (Pierre Robin manikin).

No difference could be detected in the ‘time-to-intubate’ and ‘time-to-ventilate’ between the four different laryngoscope blades in the total group of 90 participants. When using the two videolaryngoscope blades, a significantly faster time to visualize the vocal cords and a significantly better CL-classification could be achieved compared to the conventional Miller or Macintosh blade. In the direct comparison, the GLP was significantly superior concerning the ‘time-to-vocal-cords’ and the CL-classification to the GSM. Regarding the oral-soft-tissue-trauma, the GLP was superior to the three other devices. Oral-soft-tissue-trauma in infants and dental trauma in older children and adults is a relevant complication during airway management. In infants the rate of tracheal intubation-associated events (TIAEs) during intubation including oral-soft-tissue-trauma occur frequently in up to 15%-20% [11]. In this study, we were able to show that there is a highly significant less oral-soft-tissue-trauma when using the hyperangulated GLP blade compared to the two conventional blades and the GSM blade.

Subjective evaluation of view and handling revealed significantly better results for the two video-based laryngoscopes compared to the conventional ones. These results could be explained by the fact that hyperangulated airway devices enable easy “view around the corner” without the need for force to align the oral, pharyngeal and tracheal axes to see the vocal cords [15, 20, 21].

These results are comparable to outcomes from a previous proprietary study comparing a Storz C-MAC^®^ Miller blade, a Verathon^®^ GLP blade and a conventional Miller blade for intubation at the TruCorp Air-Sim^®^ Pierre Robin manikin. In the GLP group, a significantly faster view on the vocal cords was detected than with the Storz C-MAC^®^Miller and a conventional Miller blade. The CL-score was significantly better with both videolaryngoscopes compared to the conventional Miller laryngoscope blade [15]. In contrast to the previously mentioned study, a potential influence of the imaging performance of the videolaryngoscope monitor (e. g. resolution or screen size) can be excluded in the present investigation because both videolaryngoscope blades used the same portable monitor (GlideScope^®^ Go^™^ handheld system). Therefore, the results reflect only the different blade shapes.

However, a certain contradiction can be seen in our results. The GLP enables a significantly faster view on the vocal cords, but the placement of the tube poses challenges for some participants, which can lead to failed attempts due to prolonged intubation times.

Our results stand in line with current reviews and studies, which indicate that in videolaryngoscopy with a hyperangulated blade passage through the glottis and thus successful intubation is often more difficult, despite an excellent view of the vocal cord level [7, 11, 15].

This has been partly attributed to technical difficulties in handling unfamiliar hyperangulated blades and can lead to a lower first-attempt success rate [7, 11, 15, 22].

Zhang investigated the success of intubation with a hyperangulated GlideScope^®^ blade in 187 patients less than 6 years of age, of whom 35% had a history of difficult intubation. They described a first-attempt rate of 80% and a total intubation success rate of 98%. However, technical difficulties in placing the ETT were described in 58% of the cases [22].

In the subgroup analysis of our study, clinical experience seems to contribute to a faster intubation using the GSM and the ML blade. With the curved GLP and the conventional Macintosh blade, there was no difference between extensive and limited clinical experienced users. The number of optimization maneuvers was significantly higher in the subgroup of limited experienced users. Our data and the published literature described above might indicate that the use of the hyperangulated blades enables an excellent view on the glottis, however, requires certain skills for placing the ETT. Correct handling and thus safe intubation with the GLP may rely less on experience than the other blades and seems to depend more on specific training on the handling of hyperangulated blades.

Saracoglu compared two conventional laryngoscope blades (Miller and Macintosh) with two videolaryngoscopes (C-MAC^®^ Miller and McGrath^™^ curved blade) during intubation of a simulated difficult pediatric airway manikin. Significantly shorter intubation times, a better view of the glottis and lower severity of dental trauma could be achieved with the C-MAC^®^ Miller videolaryngoscope [23].

In the here presented study, only the GSM led to an intubation rate of 100%. These results are comparable to a study by Vlatten et al. [24], in which 20 anesthesia residents performed tracheal intubation on a PRS manikin with a conventional Miller blade and a C-MAC^®^ Miller videolaryngoscope blade. They found a successful intubation in 96% with the conventional laryngoscope and 100% with the videolaryngoscope. There was no difference in the ‘time-to-intubate’ between the two devices. Videolaryngoscopy provided a significantly better view on the vocal cords [24]. A better view might be clinically important to improve the first-pass success rate and to reduce adverse events like unnoticed esophageal intubation.

In our current study there were no esophageal intubations with the videolaryngoscopes, whereas there were five esophageal intubations with the conventional blades (MC: 3; ML: 2), which only became apparent when the ventilation was detected. These results are in line with the results of Garcia-Marcinkiewicz and colleagues [18]. In a randomized multicenter study on the first-attempt success rate of videolaryngoscopy in small infants, the use of a videolaryngoscope resulted in significantly fewer esophageal false intubations (videolaryngoscopy <1%, direct laryngoscopy 3%) [18].

Videolaryngoscopes with hyperangulated blades can improve intubation success in a simulated model of a difficult airway in adult patients [25]. When managing the simulated difficult pediatric airway, however, videolaryngoscopes with a straight Miller blade appear to be better suited for successful intubation [15, 23]. In this current study we found a prolonged intubation time (>120s) in six cases exclusively when using the GLP. Although the GLP provided a significantly faster view of the glottis, a significantly better CL- score and a significantly lower oral-soft-tissue-trauma, the lowest rate of successful intubations (93.3%) was recorded using the GLP. The curvature seems to ensure a better view on one side. Significant difficulties in tube placement and prolonged intubation times appear to be the downside of the hyperangulation. Desai and colleagues compared the intubation success of the Airtraq^™^ and the GlideScope^®^ videolaryngoscope in a PRS manikin. Both devices have hyperangulated blades. They found that the duration of the first intubation attempt, the duration of the first intubation success, the number of necessary intubation attempts and the number of successful intubations performed in under 30s and under 60s were significantly superior with the Airtraq^™^. Especially the tube advancement was preferentially rated in the Airtraq^™^ [26]. These results may be attributed to the Airtraq’s^™^ tube routing, where the tube is guided in a channel on the device to the vocal cord plane without the need for a stylet. When using the GlideScope^®^, a prebent stylet is required. In our study, in almost 39% of the cases, the preformed stylet was deformed after intubation, indicating difficulties in tube placement. This can lead to prolonged or even impossible intubation. Consistent with Sohn et al., we believe that the use of non-standard hyperangulated videolaryngoscope blades requires a different technique for both direct laryngoscopy and videolaryngoscopy. The lack of adequate experience or training may lead to a failure in securing the airway [19]. Despite these worrying and potentially patient-endangering intubation times, most participants rated the GLP as their device of choice in our study. The good and fast view may give the user a false sense of security and plays a major role in the retrospective assessment.

This might be a starting point for future studies, on how the better and faster view with videolaryngoscopes with hyperangulated blades can be converted into a faster and more successful intubation success through appropriate training of eye-hand coordination on pediatric difficult airway manikins in experienced and unexperienced users.

Prolonged intubation attempts in newborns and infants are associated with an increased rate of perioperative complications [2, 3, 5]. Physicians must be educated and trained specifically in airway management in high-risk patient groups [11]. In a clinical setting, this is neither feasible nor ethically justifiable, so basic training should be carried out with suitable airway trainers, such as the PRS manikin used in this study. Videolaryngoscopy can aid as a tool for teaching airway management since the trainee and the teacher share the same view of the airway [24]. In this case, the video-based Miller blade plays a special role, since it can be used as a conventional laryngoscope by the trainee, while the trainer can follow the progress of the intubation on the display, give instructions and intervene in case of doubt. According to the literature this approach can significantly increase intubation success rate from 33% to 57% and from 41% to 66%, respectively [27, 28]. However it needs to be clearly emphasized that children with PRS like any other children with expected difficult airway management should be treated by experienced personnel [24].

### Limitations

This study has some limitations: Intubations were performed on a commercially available PRS manikin and not on patients. However, according to the manufacturer, the Air-Sim^®^ Pierre Robin X manikin has been designed according to real computed tomography data and represents the anatomically correct airway of an infant with PRS. Therefore, manikins like this are frequently used for airway training and studies. However, training on a manikin can never fully reflect a life-like experience. Manikins like this have been used in several investigations before [15, 21, 23, 24, 26]. Studies on patients are limited due to the small incidence of PRS and ethical aspects. The study methodology does not allow blinding and includes subjective ratings of participants and examiners with consecutive risk of bias. Despite a randomized order of the intubation devices, an individual learning effect cannot be ruled out with certainty. Working with all the blades and especially with the traditional blades used in this study is part of the participants’ daily routine and is embedded in the standard of care at our clinic. We could not rule out the possibility that very specific training during the continuing education could improve the operator skills with a single device. In addition, the participants were aware that their performance was being assessed, which could have led to altered performance. We used a malleable stylet to facilitate endotracheal intubation with every blade. We deliberately refrained from using a rigid style, as this is not standard of our daily clinical practice. Furthermore, Turkstra and colleagues were able to prove that the use of a rigid style showed no advantages to the normal malleable stylet even in unexperienced and experienced users [29, 30].

## Conclusions

When securing the difficult airway in neonates and infants, the use of videolaryngoscopy leads to a significantly decreased time to visualize the vocal cords and to a significantly better CL-classification. Videolaryngoscopy prevents incorrect esophageal intubations. Videolaryngoscopy with a hyperangulated blade revealed a highly significant lower oral-soft-tissue-trauma compared to the other studied devices. However, a fast and good view of the vocal cords with hyperangulated videolaryngoscope blades can give the user a false sense of security due to the more difficult and time-consuming placement of the tube. In the clinical setting of difficult airway in neonates and infants with PRS videolaryngoscopy using a Miller blade seems to be advantageous in terms of successful intubation to the use of conventional direct laryngoscopy and videolaryngoscopy with hyperangulated blades and guarantees increased security. Future studies should examine whether specific training can convert the better and faster visualization of the vocal cords and the less oral-soft-tissue-trauma with the hyperangulated videolaryngoscope blade into a faster and higher intubation success rate in pediatric patients.

## Supporting information

S1 Dataset Database for statistical analysis. (SAV)
